# Prognostic factors in the surgical treatment of non-functioning pituitary adenomas: a retrospective cohort study

**DOI:** 10.20945/2359-4292-2026-0039

**Published:** 2026-04-01

**Authors:** Ana Clara Toschi Aquno, João Lucas Gomes Salgado, Lucas dos Santos Borgatto, Pedro Tadao Hamamoto Filho, José Vicente Tagliarini, Marco Antonio Zanini, Vania dos Santos Nunes Nogueira, Adriano Yacubian-Fernandes

**Affiliations:** 1 Universidade Estadual Paulista, São Paulo, SP, Brasil; 2 Universidade de São Paulo, São Paulo, SP, Brasil

**Keywords:** Pituitary neoplasms, nonfunctioning pituitary adenoma, transsphenoidal surgerypostoperative complications, prognostic factors

## Abstract

**Objective:**

This study aimed to analyze preoperative, intraoperative, and postoperative factors that affect the surgical prognosis of non-functioning pituitary adenomas (NFPA) and to determine postoperative complication rates.

**Materials and methods:**

We conducted a retrospective cohort study of patients with NFPA who underwent surgery between 1995 and 2024 at a tertiary public hospital in Brazil. Variables analyzed included tumor size, cavernous sinus invasion (Knosp classification), endocrinological status, preoperative clinical features, surgical complications, and outcomes. Statistical significance was set at *p* ≤ 0.05.

**Results:**

Seventy-three patients were included, with a mean age of 53 years and a slight predominance of females. Tumors were classified as macroadenomas (59%) and giant adenomas (41%). The transsphenoidal approach was used in 81% of cases. Partial resection was achieved in 56%, subtotal in 16%, and total in 27%. Immediate postoperative complications included diabetes insipidus (30%), bleeding (11%), hydrocephalus (10%), ischemia (10%), meningitis (6%), and cerebrospinal fluid fistula (11%). Mortality was 9.6%, significantly associated with postoperative hydrocephalus, ischemia, and larger tumor size. Giant tumors were correlated with higher rates of preoperative neurological deficits and postoperative complications. Transcranial surgery was more frequently performed in cases of giant adenomas and was associated with increased rates of ischemia and neurological deficits. Tumor recurrence was observed in 33% of patients over a mean follow-up of 48 months. Tumor size and postoperative complications such as hydrocephalus and ischemia were associated with increased morbidity and mortality.

**Conclusion:**

Postoperative hydrocephalus, ischemia, and tumor size are key determinants of mortality in NFPA surgical treatment. Implementing preventive and management strategies targeting these complications could improve patient outcomes, albeit rigorous long-term follow-up is essential due to the high rates of recurrence and reoperation.

## INTRODUCTION

Non-functioning pituitary adenomas (NFPA) account for 22%-54% of all pituitary adenomas and are characterized by the absence of clinical signs of hormonal hypersecretion ^(1)^. The main symptoms of NFPA result from mass effects, such as visual impairment from optic chiasm compression, headache ^(2,3)^, and apoplexy ^(4,5)^, as well as hormonal deficiencies caused by compression of the adenohypophysis ^(6,7)^. Because of their indolent growth and lack of overt endocrine hypersecretion, NFPAs are often diagnosed at a late stage, frequently when tumors have reached substantial size ^(8)^.

The preferred treatment for NFPA is transsphenoidal surgery via the endoscopic approach with microscopy, which offers higher rates of tumor resection ^(1,9,10)^. Multiple clinical, radiological, and surgical variables have been proposed as predictors of prognosis. Tumor size, cavernous sinus invasion, and extent of resection are consistently highlighted as primary determinants of recurrence risk. Giant non-functioning adenomas (>4 cm) pose greater surgical challenges, require specific approaches, and are associated with higher morbidity ^(11,12)^. Although gross total resection is associated with better tumor control, it may also increase the risk of new hypopituitarism or surgical complications. The postoperative period is crucial for patient quality of life, which emphasizes the need to evaluate factors influencing surgical prognosis and associated complications ^(13,14)^.

The surgical prognosis of pituitary adenomas is influenced by various clinical factors, including age, tumor size, invasiveness, hormonal activity, and the extent of resection. Transsphenoidal surgery remains the standard approach, with gross total resection resulting in improved outcomes and lower recurrence rates. Nevertheless, recurrence may still occur, particularly in non-functioning adenomas or those with cavernous sinus invasion ^(15)^. Although researchers have addressed outcomes after NFPA surgery ^(16,17)^, most have emphasized recurrence or long-term tumor control, with factors directly associated with surgical success being less clearly defined.

To address this gap, this retrospective cohort study aimed to analyze the main factors related to surgical success of non-functioning pituitary adenoma treatment. Specifically, we sought to identify preoperative, intraoperative, and postoperative predictors of complete resection and functional outcomes, as well as to determine the rate of surgical complications in relation to clinical and surgical variables.

## MATERIALS AND METHODS

### Study design and setting

This retrospective cohort study is reported in accordance with the STROBE (Strengthening the Reporting of Observational Studies in Epidemiology) guidelines ^(18)^. Data were obtained from the Neurosurgery Service at Hospital das Clínicas, Faculty of Medicine of Botucatu, a tertiary referral center in Brazil. The study was approved by the Research Ethics Committee of the same institution (CAAE no. 71548323.4.0000.5411). Given its retrospective nature, the requirement for informed consent was waived in accordance with national regulations.

### Participants

We reviewed the medical records of all patients diagnosed with NFPA and who underwent surgical treatment between 1995 and 2024.

### Variables

The following variables were extracted and analyzed:

- Preoperative imaging findings: tumor size and cavernous sinus invasion, with the latter classified according to the Knosp classification ^(19)^.
- Endocrinological assessment: including the presence of panhypopituitarism.
- Preoperative clinical presentation: with emphasis on visual loss.
- Surgical complications: including cerebrospinal fluid fistula, meningitis, diabetes insipidus, worsening of visual status, and bleeding in the tumor bed.
- Unfavorable surgical outcomes: defined as incomplete tumor resection or procedure-related mortality.

### Data sources and measurement

All data were obtained from institutional medical records. Data extraction was performed by trained researchers using a standardized protocol to ensure consistency.

### Bias and study size

As a retrospective study, potential sources of bias included variability in the completeness of medical records and the extended follow-up period. To mitigate this, only patients with complete preoperative, surgical, and postoperative data were included.

### Statistical analysis

Categorical variables were summarized as absolute and relative frequencies, and comparisons were made using the chi-square or Fisher’s exact test, as appropriate. A *p*-value of ≤ 0.05 was considered statistically significant. Analyses were performed using Statistical Package for the Social Sciences (SPSS), version 24.0, for MacBook (IBM Corp., Armonk, NY, USA).

## RESULTS

### Participants and descriptive data

**Figure 1:**
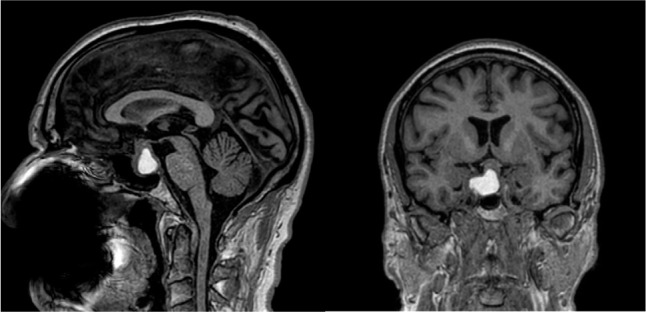
Sagittal and coronal views of T1 MRI without gadolinium showing an apoplexy of a pituitary macroadenoma.

A total of 73 patients met the eligibility criteria and were included in the analysis (**Figure 1**). The mean age was 52.99 years (standard deviation [SD] ±13.41). Of these, 39 were women (53.4%) and 34 were men (46.6%). Regarding race, 60 patients (82.2%) were white, 10 (13.7%) were brown, and 3 (4.1%) were black. Comorbidities included systemic arterial hypertension in 27 patients (37.0%), diabetes mellitus in 8 (11.0%), dyslipidemia in 14 (19.2%), smoking in 16 (21.9%), and alcoholism in 6 (8.2%).

### Preoperative clinical characteristics

Preoperative neurological conditions included oculomotor nerve (cranial nerve III) paresis in 4 patients (5.5%), hydrocephalus in 4 (5.5%), mental confusion in 3 (4.1%), and headache in 28 (38.4%). Visual complaints were frequent, with hemianopsia reported in 49 patients (67.1%) and amaurosis in 16 (21.9%). Pituitary apoplexy and menstrual alterations were each observed in 10 patients (13.7%). **Figure 1** shows pituitary apoplexy on a T1-weighted magnetic resonance image.

### Preoperative endocrinological status

Endocrinological assessment revealed hyperprolactinemia (based on diluted prolactin measures) in 16 patients (21.9%), panhypopituitarism in 28 (38.4%), hypocortisolism in 13 (17.8%), hypogonadism in 18 (24.7%), and hypothyroidism in 25 (34.2%).

### Tumor characteristics

**Figure 2:**
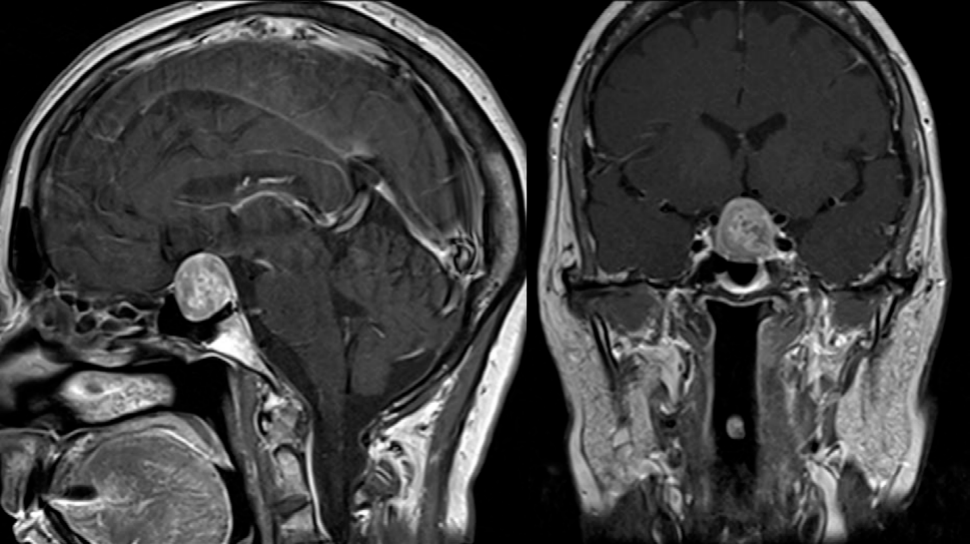
Sagittal and coronal views of T1 MRI with gadolinium showing a pituitary macroadenoma.

**Figure 3:**
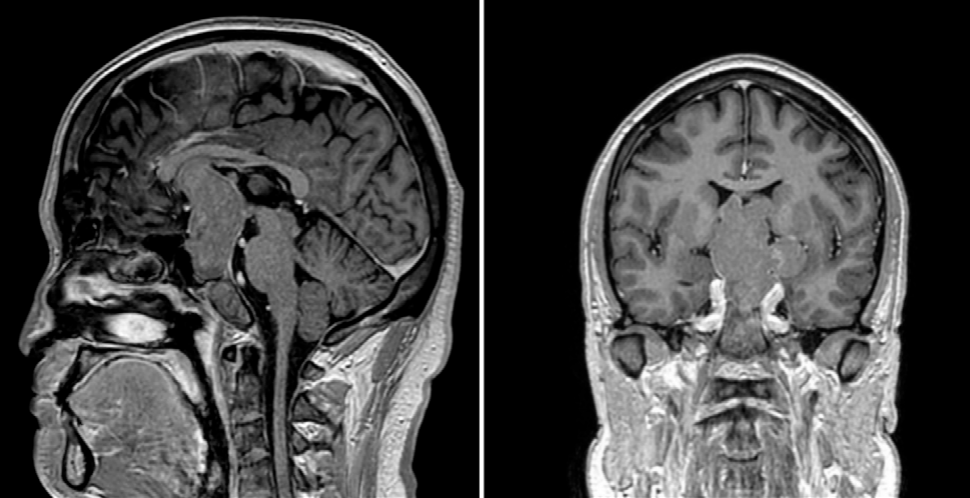
Sagittal and coronal views of T1 MRI with gadolinium showing a pituitary giant adenoma.

Of the cohort, 43 patients (58.9%) had macroadenomas and 30 (41.1%) had giant adenomas. Cavernous sinus invasion was graded by the Knosp classification: grade 1 in 8 tumors (11.0%), grade 2 in 16 (21.9%), grade 3a in 12 (16.4%), grade 3b in 2 (2.7%), and grade 4 in 18 (24.7%). **Figure 2** shows a macroadenoma, while **Figure 3** depicts a giant adenoma of the pituitary gland.

### Surgical approach and extent of resection

The primary surgical approach was transsphenoidal, performed in 59 patients (80.8%). Transcranial surgery was conducted in 14 cases (19.2%), comprising 6 pterional, 7 fronto-orbitozygomatic, and 1 transcallosal approach. The extent of tumor resection was partial in 41 patients (56.2%), subtotal in 12 (16.4%), and total in 20 (27.4%).

### Postoperative complications

Immediate postoperative complications included diabetes insipidus in 22 patients (30.1%), syndrome of inappropriate antidiuretic hormone secretion in 6 (8.2%), tumor bed bleeding in 8 (11.0%), hydrocephalus in 7 (9.6%), meningitis in 4 (5.5%), cerebral ischemia in 7 (9.6%), and cerebrospinal fluid fistula in 8 (11.0%). Persistent diabetes insipidus was observed in 12 patients (16.4%), and postoperative panhypopituitarism developed in 26 (35.6%).

### Functional outcomes and late deficits

Visual improvement was observed in 36 patients (49.3%). Late postoperative neurological deficits included 5 patients (6.8%) becoming bedridden, 2 (2.7%) experiencing visual deterioration, 2 (2.7%) with hemiparesis, and 2 (2.7%) developing dementia.

### Follow-up and long-term outcomes

During a mean follow-up of 48 months (range: 3-276 months), tumor recurrence occurred in 24 patients (32.9%), reoperation was required in 14 (19.2%), and radiotherapy was administered in 5 (6.8%). There were 7 deaths recorded, corresponding to a mortality rate of 9.6%. **Table 1** systematically presents these results. Among significant statistical associations, mortality was associated with postoperative hydrocephalus (*p* < 0.001), postoperative ischemia (*p* = 0.012), and tumor size (*p* = 0.017), with higher mortality among patients with giant tumors. Tumor size significantly correlated with preoperative oculomotor paresis (*p* = 0.05), preoperative hydrocephalus (*p* = 0.05), and preoperative hyperprolactinemia (*p* = 0.048), being lower in giant adenomas compared to macroadenomas. Transcranial surgery was associated with giant tumors (*p* = 0.015), postoperative ischemia (*p* = 0.022), and postoperative deficits (*p* = 0.031). The occurrence of postoperative cerebrospinal fluid fistula was associated with meningitis (*p* = 0.003). **Table 2** lists these significant statistical associations.

**Table 1:** General results

Parameter	Result
Number of patients evaluated (n)	73
Mean age (years)	52.99 ± 13.41
Sex (n, %)	
Women	39 (53.4%)
Men	34 (46.6%)
Race (n, %)	
White	60 (82.2%)
Brown	10 (13.7%)
Black	3 (4.1%)
Comorbidities (%)	
Hypertension	37.0%
Diabetes mellitus	11.0%
Dyslipidemia	19.2%
Smoking	21.9%
Alcoholism	8.2%
Preoperative neurological conditions (%)	
Oculomotor nerve paresis	5.5%
Hydrocephalus	5.5%
Mental confusion	4.1%
Headache	38.4%
Visual complaints (%)	
Hemianopsia	67.1%
Amaurosis	21.9%
Other preoperative symptoms (%)	
Pituitary apoplexy	13.7%
Menstrual alterations	13.7%
Preoperative endocrinological status (%)	
Hyperprolactinemia	21.9%
Pan-hypopituitarism	38.4%
Hypercortisolism	17.8%
Hypogonadism	24.7%
Hypothyroidism	34.2%
Tumor size classification (%)	
Macroadenomas	58.9%
Giant adenomas	41.1%
Cavernous sinus invasion (Knosp classification) (%)	
Grade 1	11.0%
Grade 2	21.9%
Grade 3a	16.4%
Grade 3b	2.7%
Grade 4	24.7%
Surgical approach (%)	
Transsphenoidal	80.8%
Transcranial	19.2%
Types of transcranial approaches (n)	
Pterional	6
Fronto-orbitozygomatic	7
Transcallosal	1
Tumor resection extent (%)	
Partial	56.2%
Subtotal	16.4%
Total	27.4%
Immediate postoperative complications (%)	
Diabetes insipidus	30.1%
Syndrome of inappropriate antidiuretic hormone secretion	8.2%
Bleeding	11.0%
Hydrocephalus	9.6%
Meningitis	5.5%
Ischemia	9.6%
Fistula	11.0%
Persistent complications (%)	
Persistent diabetes insipidus	16.4%
Postoperative panhypopituitarism	35.6%
Visual improvement (n, %)	36, 49.3%
Late postoperative deficits (%)	
Bedridden	6.8%
Visual worsening	2.7%
Hemiparesis	2.7%
Dementia	2.7%
Late follow-up outcomes (%)	
Tumor recurrence	32.9%
Reoperation	19.2%
Radiotherapy	6.8%
Mortality (n, %)	7, 9.6%
Mean follow-up duration	48 months (range: 3-276 months)

**Table 2:** Significant statistical associations

Variable	Mortality		*p*-value
	Mortality (yes)	Mortality (no)	
Hydrocephalus (postoperative) (yes)	5 (71.4%)	2 (28.6%)	< 0.001
Hydrocephalus (postoperative) (no)	2 (3.3%)	59 (96.7%)	
Ischemia (postoperative) (yes)	3 (50%)	3 (50%)	0.012
Ischemia (postoperative) (no)	4 (6.5%)	58 (93.5%)	
Tumor size (giant adenomas)	6 (21.4%)	22 (78.6%)	0.017
Tumor size (macroadenomas)	1 (2.5%)	39 (97.5%)	
Variable	Surgery type		*p*-value
	Transsphenoidal	Transcranial	
Tumor size (macroadenomas)	39 (90.7%)	4 (9.3%)	0.015
Tumor size (giant adenomas)	20 (66.7%)	10 (33.3%)	
Ischemia (no)	56 (94.9%)	10 (71.4%)	0.022
Ischemia (yes)	3 (5.1%)	4 (28.6%)	
Postoperative deficit (no)	53 (89.85)	9 (64.3%)	0.031
Postoperative deficit (yes)	6 (10.15%)	5 (35.7%)	
Variable	Meningitis		*p*-value
	Meningitis (no)	Meningitis (yes)	
Cerebrospinal fluid fistula (no)	64 (98.5%)	1 (1.5%)	0.003
Cerebrospinal fluid fistula (yes)	5 (62.5%)	3 (37.5%)	

## DISCUSSION

This study analyzed 73 patients with NFPA who underwent surgery, identifying key determinants of prognosis and outcomes. The cohort included 59% macroadenomas and 41% giant adenomas, with the transsphenoidal approach used in 81% of cases. Partial resection was most frequent (56%), and postoperative complications were common, including diabetes insipidus (30%), hydrocephalus (9.6%), ischemia (9.6%), cerebrospinal fluid fistula (11%), and meningitis (5.5%). The mortality rate was 9.6%, significantly associated with postoperative hydrocephalus, ischemia, and larger tumor size. Giant adenomas were linked to higher rates of preoperative neurological deficits and postoperative complications; transcranial surgery was also more frequent in these patients and associated with increased ischemia and neurological deficits. Tumor recurrence occurred in 33% of cases over an average follow-up of 48 months. These data underscore tumor size and severe postoperative complications as critical determinants of surgical prognosis in NFPA.

These results provide a comprehensive analysis of the demographic, clinical, neurological, endocrinological, and tumor characteristics, as well as surgical interventions, postoperative complications, and outcomes of patients with non-functioning pituitary macroadenomas. The findings corroborate the need for guidelines that emphasize preoperative endocrinological evaluation even in silent NFPA ^(8)^, ophthalmological assessment ^(20)^, complex surgical approaches when needed ^(21,22)^, and rigorous postoperative follow-up of these patients ^(23,24)^.

The results provide a comprehensive view of non-functioning pituitary macroadenomas, highlighting clinical and surgical patient characteristics, immediate and late complications, and outcomes, thereby enhancing our understanding of the condition and guiding clinical and surgical management. Evidence has pointed to high morbidity and mortality in NFPA surgery, evidencing prognostic factors such as cerebrovascular disease ^(25)^, prolonged glucocorticoid use ^(26)^, panhypopituitarism ^(27,28)^, and patient age ^(29)^. Females and younger patients ^(30)^, as well as tumor size ^(31)^, have also been associated with higher morbidity and mortality. Similarly, researchers have indicated predictors of postoperative evolution in these tumors regarding tumor recurrence (resection degree and Knosp classification) ^(32)^, cerebrospinal fluid fistula (tumor size and local invasion) ^(33)^, and visual recovery (integration of clinical and imaging findings) ^(34)^.

The occurrence of hydrocephalus has been reported in some studies ^(35,36)^, and in our series, postoperative hydrocephalus showed a strong association with mortality. Hydrocephalus may result from cerebrospinal fluid obstruction due to surgery or tumor mass itself, leading to increased intracranial pressure and severe neurological complications. This observation suggests that postoperative hydrocephalus identifies patients at higher risk of adverse outcomes, emphasizing the need for strict surveillance and prompt interventions to manage intracranial pressure and prevent fatal complications.

Postoperative cerebral ischemia was also significantly associated with mortality. Ischemia can result from vascular compromise during surgery, especially in cases with large tumors or cavernous sinus invasion, leading to serious neurological deficits and poorer prognosis ^(25)^. This finding highlights the importance of meticulous surgical planning and intraoperative management to minimize ischemic risk, as along with intensive neurological monitoring after surgery ^(12)^.

Mortality was higher in patients with giant adenomas compared to those with macroadenomas. Larger tumors present greater surgical complexity, more extensive invasion of adjacent structures (as seen in the high grades of Knosp cavernous sinus invasion classification), and increased risk of postoperative complications (e.g., hydrocephalus and ischemia). Tumor size, particularly with suprasellar extension, is a relevant prognostic factor, associating not only with mortality but also with additional severe complications ^(31,37,38)^.

Apart from mortality, tumor size was significantly associated with preoperative oculomotor nerve paresis (*p* = 0.05), preoperative hydrocephalus (*p* = 0.05), and preoperative hyperprolactinemia (*p* = 0.048). Notably, hyperprolactinemia, as reported in NFPA ^(39)^, occurred less frequently in giant adenomas than in macroadenomas, possibly due to differences in functional activity or pituitary tissue compression. These findings indicate that larger tumors exert a greater neurological and structural impact before surgery, reflecting their aggressiveness and extent.

Transcranial surgery was performed more frequently in giant tumors (*p* = 0.015) and was associated with a higher incidence of ischemia (*p* = 0.022) and postoperative deficits (*p* = 0.031). This reinforces the greater technical challenge and higher risks inherent in more invasive approaches, which are required for larger and more extensive tumors ^(22)^. The elevated risk of neurological and ischemic complications contributes to increased mortality and morbidity in this subgroup.

Postoperative cerebrospinal fluid fistula ^(33)^ was significantly associated with meningitis, a serious complication that can negatively impact recovery and increase mortality risk. This finding emphasizes the need for surgical techniques that minimize the incidence of fistulas and for stringent protocols to prevent and manage infections.

Statistically significant associations revealed that mortality in patients with non-functioning pituitary macroadenomas is strongly related to severe postoperative neurological complications such as hydrocephalus and ischemia and tumor size, which influences surgical complexity and the risk of such complications. This evidence indicates that the management of these patients must prioritize careful risk assessment, intensive postoperative monitoring to detect complications such as hydrocephalus and ischemia early, and strategies to minimize harm, especially in cases of giant tumors. Additionally, the association between cerebrospinal fluid fistula and meningitis reinforces the importance of preventing infectious complications, which significantly worsen prognosis and increases mortality. Given the high rates of tumor recurrence and reoperation, long-term follow-up must be integral to patient management ^(23,24,32)^. Conservative treatment of NFPA is reserved for small, asymptomatic tumors that must be closely monitored through imaging and endocrinological controls ^(40,41)^. After all, the smaller the tumor, the better the surgical outcome.

The main limitations of this study include its retrospective, single-center design, which may introduce selection bias and limit generalizability. Additionally, the relatively small sample size of 73 patients over an extended period (1995-2024) may have affected the statistical power for detecting certain associations. Heterogeneity in surgical approaches and advances in techniques over nearly three decades may have also impacted outcomes and were not fully controlled for. Some postoperative complications and long-term outcomes may have also been underreported due to variable follow-up durations (ranging from 3 to 276 months). Collectively, these factors may have restricted the ability to draw definitive conclusions regarding prognostic factors in non-functioning pituitary adenoma surgery.

The long-term follow-up (mean, 48 months; range: 3-276 months) allowed detailed assessment of tumor recurrence and late complications, which is critical given the indolent nature and late diagnosis of NFPAs. This study’s comprehensive evaluation of clinical, endocrinological, radiological, and surgical factors over nearly three decades at a tertiary referral center provides valuable institutional data on surgical outcomes and risk factors in a Brazilian population, which may have unique epidemiological characteristics. The inclusion of a relatively large and demographically diverse cohort with detailed complication assessment reinforces the value of this report as it addresses knowledge gaps in perioperative predictors of surgical success and mortality in NFPA patients.

Clinically, identifying tumor size and severe postoperative complications such as hydrocephalus and ischemia as key predictors of mortality can improve preoperative risk stratification. These findings support intensifying perioperative monitoring, especially in patients with giant adenomas, to rapidly detect and address complications (e.g., hydrocephalus and ischemia), potentially reducing morbidity and mortality. Preventive measures targeting cerebrospinal fluid fistula formation and meningitis should be emphasized in surgical and postoperative care protocols. Early diagnosis through endocrinological and ophthalmological evaluation may facilitate detection of smaller tumors, which are associated with better surgical outcomes, advocating for improved screening strategies. Moreover, our findings underscore the need for individualized surgical planning, balancing gross total resection against the risk of hypopituitarism and complications, especially when considering transcranial approaches for large tumors. Long-term follow-up is essential due to high recurrence and reoperation rates, influencing postoperative surveillance guidelines. In summary, this study advances understanding of NFPA by detailing how tumor size and critical postoperative complications influence mortality and surgical outcomes, and underscores refined perioperative risk assessment, preventive strategies for complications, and early detection to improve clinical management and patient prognosis.

Regarding management protocols for complications, our approach to cerebrospinal fluid fistula involved prompt identification through clinical and imaging evaluation, followed by initial conservative measures, including bed rest and head elevation. If conservative management failed, surgical repair was performed using multilayer reconstruction techniques with autologous tissue grafts, with lumbar drainage reserved for persistent cases. Postoperative hydrocephalus was managed by close neurological monitoring and imaging, with ventricular drainage or ventriculoperitoneal shunting employed in patients with symptomatic hydrocephalus or increased intracranial pressure. Cerebral ischemia was addressed via intensive neurological monitoring and supportive care, including optimization of cerebral perfusion and prevention of secondary insults, while surgical planning aimed to minimize vascular injury, particularly in cases with cavernous sinus invasion. Diabetes insipidus was managed with fluid and electrolyte monitoring, desmopressin as needed, and patient education, with persistent diabetes insipidus cases followed long-term with endocrinological support.

Over the years, preventive strategies have included adopting endoscopic transsphenoidal surgery (used in 81% of cases) due to its superior visualization, aiding tumor resection and skull base reconstruction, complemented by microsurgical techniques. To prevent cerebrospinal fluid fistula leaks, multilayer closure methods were employed, including vascularized nasoseptal flaps when feasible, fat grafts, and synthetic dural substitutes. Prophylactic lumbar drainage was selectively applied in high-risk cases with extensive dural defects or intraoperative cerebrospinal fluid fistula leaks. Rigorous intraoperative hemostasis and careful vascular management aimed to reduce ischemic complications. Postoperative monitoring protocols have been established to identify early signs of hydrocephalus and diabetes insipidus.

Lessons learned and recommendations for future practice stress the importance of early diagnosis through comprehensive endocrinological and ophthalmological evaluation, enabling earlier treatment of smaller tumors and potentially reducing complication rates. Since giant adenomas requiring transcranial approaches were associated with more ischemic and neurological deficits, minimally invasive endoscopic approaches should be prioritized whenever possible. Advances in reconstruction techniques, including adoption of vascularized flaps and meticulous multilayer reconstruction, have become central to reducing cerebrospinal fluid fistula and subsequent meningitis. Intensive postoperative monitoring with standardized protocols including neuroimaging and neurological assessments is critical for the early detection and management of hydrocephalus and ischemia, thereby decreasing mortality. Multidisciplinary care involving neurosurgeons, endocrinologists, and otolaryngologists is essential for optimizing patient outcomes. Although the retrospective nature and evolving surgical techniques over the 29-year period may have influenced complication rates, these insights form the basis for ongoing refinement of our protocols. Hence, we aim to incorporate these management approaches, preventive strategies, and lessons learned to provide a more comprehensive and instructive discussion for the readership.

In conclusion, this study provides a solid foundation for understanding factors influencing mortality in non-functioning pituitary macroadenomas, highlighting postoperative hydrocephalus, ischemia, and tumor size as key elements. Strategies focused on preventing and managing these complications may contribute to reducing mortality and improving clinical outcomes in these patients. Early diagnosis of these tumors through endocrinological and ophthalmological diagnosis confirmed by imaging exams enables detection of smaller tumors and more favorable surgical treatment results.

## Data Availability

data from the ELSA-Brasil Study is subject to restricted access.
